# Physiology Responses and Players’ Stay on the Court During a Futsal Match: A Case Study With Professional Players

**DOI:** 10.3389/fpsyg.2020.620108

**Published:** 2020-12-14

**Authors:** Julio Wilson Dos-Santos, Henrique Santos da Silva, Osvaldo Tadeu da Silva Junior, Ricardo Augusto Barbieri, Matheus Luiz Penafiel, Roberto Nascimento Braga da Silva, Fábio Milioni, Luiz Henrique Palucci Vieira, Diogo Henrique Constantino Coledam, Paulo Roberto Pereira Santiago, Marcelo Papoti

**Affiliations:** ^1^Laboratory and Research Group on Physiology Applied to Sports Training (FITES), Department of Physical Education, School of Sciences, São Paulo State University (UNESP), São Paulo, Brazil; ^2^Postgraduate Program in Movement Sciences, São Paulo State University (UNESP), São Paulo, Brazil; ^3^Federal Institute of Education, Science and Technology of São Paulo, Boituva, Brazil; ^4^School of Physical Education and Sports, University of São Paulo (USP), São Paulo, Brazil

**Keywords:** lactate, recovery, team sport, playing time, outfield players, heart rate, intensity of effort

## Abstract

Physiological responses in futsal have not been studied together with temporal information about the players’ stay on the court. The aim of this study was to compare heart rate (HR) and blood lactate concentration ([La^−^]) responses between 1-H and 2-H considering the time of permanency of the players on the court at each substitution in a futsal match. HR was recorded during entire match and [La^−^] was analyzed after each substitution of seven players. %HR_mean_ (89.61 ± 2.31 vs. 88.03 ± 4.98 %HR_max_) and [La^−^] mean (8.46 ± 3.01 vs. 8.17 ± 2.91 mmol·L^−1^) did not differ between 1-H and 2-H (ES, trivial-small). Time in intensity zones of 50–100 %HR_max_ differed only in 60–70 %HR_max_ (ES, moderate). HR coefficient of variation throughout the match was low (7%) and among the four outfield players on the court (quartets, 5%). Substitutions (2 player’s participation in each half), time of permanence on the court (7.15 ± 2.39 vs. 9.49 ± 3.80 min), ratio between time in- and out-ratio on the court (In:Outcourt = 1:1.30 ± 1:0.48 vs. 1:1.05 ± 1:0.55 min) also were similar between 1-H and 2-H (ES, moderate and small, respectively). Balancing the number of substitutions, and the In:Outcourt ratio of players in both halves of the match, playing lower time at 1-H, ~8 min for each participation in the match, made it possible to maintain intensity of the match in 2-H similar to the 1H. These results are a good guidance to coaches and for application in future studies.

## Introduction

Futsal is an adaptation of soccer for practice on the court played in two halves of 20-min with a 10-min interval between halves, with higher intensity than other team sports as soccer, handball and basketball (Barbero-Alvarez et al., 2008), demanding aerobic and anaerobic pathway. Heart rate has presented 90% of the maximum HR (%HR_max_) (Barbero-Alvarez et al., 2008) and blood lactate concentration ([La^−^]) presented values between 5 and 5.5 mmol·L^−1^ in match (Castagna et al., 2009; Makaje et al., 2012).

The high demand of the match is an indication that the players may have difficulty maintaining the intensity of effort in the second half. Professional futsal players had lower mean HR (HR_mean_) in the second half (2-H) compared to the first half (1-H), 88.1 vs. 91.1% of HR_max_, respectively, and percentage of time spent in high intensity HR zone (Barbero-Alvarez et al., 2008). Distance covered in high intensity running also decay in 2-H in official matches (Barbero-Alvarez et al., 2008; de Oliveira Bueno et al., 2014). In contrast, in a simulated match the HR did not differ among the four 10-min periods (Castagna et al., 2009) as well as parameters relationship to the players’ sprints did not differ between 1-H and 2-H in official matches of professional players (Barbero-Alvarez et al., 2008; de Oliveira Bueno et al., 2014; Caetano et al., 2015; Vieira et al., 2016), friendly match (Vieira et al., 2016) and simulated match (Milioni et al., 2016).

Reduction of [La^−^] after 2-H in universities friendly match (Tessitore et al., 2008) and amateur friendly match (Antunes Neto et al., 2007) has been reported in futsal. However, in simulated match with four 10-min periods the [La^−^] remained unchanged, with a mean of 5.3 mmol·L^−1^ (Castagna et al., 2009). Anaerobic metabolism can have an important role to energy provision during the match, however, in studies with team sports the blood samples are collected regularly at the end of half time and it can result in loss of information (Stolen et al., 2005). Since substitutions are unlimited in futsal, to verify players’ [La^−^] after each substitution during the entire futsal match provides more accurate information for understanding the anaerobic demand. In addition, analyzing the timing of players on the court, as well as the rest time between substitutions, can provide important information for understanding the reduction of physiological responses in 2-H. For example, in soccer it is well established that the HR (Dellal et al., 2012), [La^−^] as well as distance covered at high intensity (Stolen et al., 2005) are reduced in the second half. However, substitutes entering the 2-H presented higher HR_mean_ and time > 90% HR_max_ (Coelho et al., 2012) and covered a greater distance at higher intensity (Bradley et al., 2014) compared to the players who started playing. Thus, the aim of this study was to compare HR and [La^−^] responses between 1-H and 2-H, considering the time of permanency of the players on the court at each substitution, in a friendly futsal match. Our hypothesis is that if there is a balance in the time the players stay on the court between 1-H and 2-H, the physiology responses can be similar between 1-H and 2-H.

## Materials and Methods

### Participants

Professional futsal players from a team competing in the Paulista League, the main competition of the State of São Paulo, Brazil, were evaluated during a friendly match, in the second week of pre-season. The players trained six times per week, 1.5 h per training session, 11 sessions per week within 6 days (two or one time per day). Of the nine outfield players evaluated, two played only in one half of the match and were excluded from the sample. Thus, seven outfield players composed the sample, two defenders, three wings and two pivots (23.1 ± 2.5 years, 174 ± 6 cm, 76.1 ± 6.6 Kg, 14.0 ± 2.9%) and intermittent aerobic capacity measured by Yo-yo test intermittent recovery level 1 (YYIR-1), 1,497 ± 299 m. The study was approved by the local Ethics Committee of the Scholl of Science of the São Paulo State University (Process 347/49/01/08), according to the laws of the country, and before its beginning, all participants were aware of the objectives and consented to their participation in data collection through a free and informed consent term.

### Proceedings

Seven players, from the State League, included in the sample were evaluated during a friendly match against a National League team. The evaluated team played in tactical system 1-3-1 (1-goalkeeper, 2-wingers, 1-defender and 1-pivot), zone marking on the defense half-court most of the match. To encourage the evaluated played, the opponent was one of the best National teams, which had already won National League, South American championships and was composed of three players from Brazil’s national team, including the best player of the world at the time. Both teams trained 2 times a week, 1–2 times/day and were in the pre-competition period.

The match was played in an official court (40 × 20 m), lasting 20 × 20 min with a 10 min interval, considering futsal official rules, included the time clocked. No guidance was given to the head coach, and his assistants on substitution procedure, substitutions were made according to the demands of the match and the strategy of the head coach. The match occurred 14 days after the start of the pre-season, physical evaluation 48 h after match, without previous exercise, and anthropometric evaluation 24 h after physical evaluation. HR was recorded during the entire match and blood samples were collected to analyze [La^−^] at each player’s substitution. In addition, %HR_max_ from four outfield players on the court playing (quartet) was determined at each substitution, considering the %HR_max_ of each quartet.

The ratio between playing and recovering time during the match was verified (In:Out_court_ ratio), considering the time players remained on the court and the subsequent out off the court recovery time, disregarding recovery time between 1-H and 2-H and recovery time after their last substitution. During the out-court period substituted players remained seated or standing, with access to water ad libitum, without ingestion of any nutritional supplement or isotonic drink.

Six authors participated in data collection, one collector for each substituted player, positioned on the side of the substitutes’ bench. Four collectors were taking the blood sample for [La^−^] analysis, another collector was monitoring HR and recording player changes in HR software, and the lead author of the study was coordinating and assisting the data collection. The match was filmed with a video camera positioned at the top of the gym, to analyze the substitutions. The characterization of the sample was made with an anthropometric evaluation, including body mass, height, body fat percentage and intermittent aerobic test.

### Anthropometric Assessment and Intermittent Aerobic Capacity

All evaluations were performed in the morning, between 9:00 and 10:30 a.m., without previous physical effort, in the week after data collection (2nd evaluation in pre-season). The stature was taken with a stadiometer fixed to the wall and the body mass with portable scale Tanita Ironman BC553 (Tanita Corporation of America Inc., Arlington Heights, IL, United States). Intermittent aerobic capacity was performed YYIR-1, which present reliability and validity (Krustrup et al., 2003). The test consists of covering the distance of 40-m (2 × 20-m shuttle runs) interspersed with a 10-s period of active recovery, with an initial speed of 10 km·h^−1^ and incremental speed to exhaustion controlled by a CD player sound signal to guide the time in which the player must be every 20-m and also the 10 s of recovery between each run of 40-m (Bangsbo, 1994). The percentage of fat was determined by Dual energy Ray-X absorptiometry, Discovery Wi model (Hologic INC, Bedford, MA, United States), with all the procedures of prior analysis and calibration of the equipment made as indicated by the manufacturer.

### Heart Rate

HR was recorded by Polar Team System 2 (Polar Electro Oy, Kempele, Fynland) every second during entire match, but the HR considered for analysis was only for the player who remained playing on the court. The HR data was recorded in the Polar Team 2 software, from the same manufacturer, and then transferred and analyzed in the Excel spreadsheet (Microsoft Office, 2010). HR_max_ was considered the highest value between match and YYIR-1 test, the HR_mean_ was expressed in absolute values and relative to %HR_max_. %HR_max_ of each quartet was determined at each substitution. The HR was also analyzed in five zones of intensity (50–60, 60–70, 70–80, 80–90, and 90–100% of HR_max_), considering the percentage of time in each %HR_max_ zone. Above 90% HR_max_ was considered as the zone of high intensity (Castagna et al., 2009).

### Blood Lactate

Samples of blood (25 μl) were taken from the athletes’ earlobe, after asepsis with alcohol, and stored in 50 μl of 1% NaF 30–45 s after the player had been substituted and at the end of first and second halves of the match. The samples were frozen and [La^−^] analysis was made in the following week with the YSL 2300 Sports lactate analyzer (YSI, OH, United States), according to manufacturer’s directions. [La^−^] mean ([La^−^]_mean_) and maximum ([La^−^]_max_) were determined considering all player’s participation on the court during the match.

### Statistical Analysis

Normality of data was tested using the Shapiro-Wilk test. Total time and time of player’s participation, HR_mean_, HR_max_, [La^−^]_mean_, [La^−^]_max_, and In:Out_court_ ratio of each period which a player remained on the court were analyzed by Student’s *t*-test for related samples, after confirming the normality. %HR_max_ and time in five of %HR_max_ did not present normal distribution and they were analyzed by nonparametric Wilcoxon signed-rank test. The difference between %HR_max_ of the four outfield players on the court (quartets) was calculated by Student’s *t*-test for unrelated samples. Data were considered from the mean of player’s participation in 1-H and 2-H to verify the difference between them, excepting HR and [La^−^] maximum values, which were taken as absolute values for each half. The Coefficient of variation (CV%) of the HR was calculated individually for each player and among the new quartets formed at each substitution, dividing the standard deviation by the mean. Data are reported as mean ± SD with 95% confidence intervals presented. Effect size (ES) was determined by Cohen’s *d* effect size for the parametric data (mean difference divided by the square root of the average of the squared standard deviations, SD_pooled_) and for nonparametric data, Wilcoxon’s test, by dividing the *Z* value by the square root of “*n*” of the two observations (*r*-value) (Cohen, 1988; Fritz et al., 2012). Threshold values for Cohen’s *d* ES index of the parametric data were considered: trivial < 0.2, small < 0.6, moderate < 1.2, large < 2.0, very large > 4.0, and to nonparametric data, considering *r*-value between 0.0 and 1.0, ES was considered: trivial < 0.1, small < 0.3, moderate < 0.5, large < 0.7, very large < 0.9, nearly perfect > 0.9, and perfect = 1.0 (Hopkins, 2002; Hopkins et al., 2009). Significance level was set at *p* < 0.05 and data was analyzed by Statistical Package for Social Sciences (SPSS) software, version 20.0 (IBM Corp. Released 2011. IBM SPSS Statistics for Windows, Version 20.0. Armonk, NY: IBM Corp).

## Results

HR_max_ was 202 ± 5 bpm (higher value between YYIR-1 and match), representing the mean of 89% of HR_max_. HR, %HR and [La^−^] were similar in 1-H and 2-H, not differing significantly in both values, mean and maximum (Table 1). Match temporal data were also not different between 1-H and 2-H (Table 1). CV% mean of the HR of the players throughout the match was 7 ± 2%, 6 ± 1% and 8 ± 2% in 1-H and 2-H, respectively.

**Table 1:** Results of the first half (1-H), second half (2-H), and match (1-H and 2-H).

	Match (95% CI)	1-H (95% CI)	2-H (95% CI)	*p*-value	ES (index)
Time of match (min)	67.00	30.28	36.72	-	-
Total time on the court (min)	31.71 ± 9.02 (23.37–40.05)	13.44 ± 5.72 (8.15–18.73)	18.19 ± 6.04 (12.60–23.77)	0.16	0.81 (moderate)
Each participation (min)	8.19 ± 2.27 (6.09–10.28)	7.15 ± 2.39 (4.94–9.35)	9.49 ± 3.80 (5.98–13.01)	0.15	0.74 (moderate)
HR_mean_ (bpm)	179 ± 6 (173–185)	181 ± 5 (176–186)	178 ± 9 (169–186)	0.19	0.43 (small)
HR_max_ (bpm)	200 ± 7 (194–206)	195 ± 9 (187–203)	198 ± 7 (192–204)	0.41	0.38 (small)
%HR_max_ (%)	88.79 ± 3.35 (85.69–91.88)	89.61 ± 2.31 (87.48–91.75)	88.03 ± 4.98 (83.42–92.63)	0.09	0.45 (small)
[La^−^]_mean_ (mmol·L^−1^)	8.32 ± 2.88 (5.65–10.98)	8.46 ± 3.01 (5.67–11.24)	8.17 ± 2.91 (5.48–10.86)	0.62	0.10 (trivial)
[La^−^]_max_ (mmol·L^−1^)	9.71 ± 3.00 (6.94–12.49)	9.16 ± 3.16 (6.23–12.08)	9.20 ± 3.14 (6.30–12.10)	0.94	0.01 (trivial)

Values expressed in mean ± SD (95% confidence interval) and difference between 1-H and 2-H. ES, effect size; HR_mean_, heart rate mean; HR_max_, maximum heart rate; %HR_max_, percentage of HR_max_; [La^−^], blood lactate concentration.

**Figure 1:**
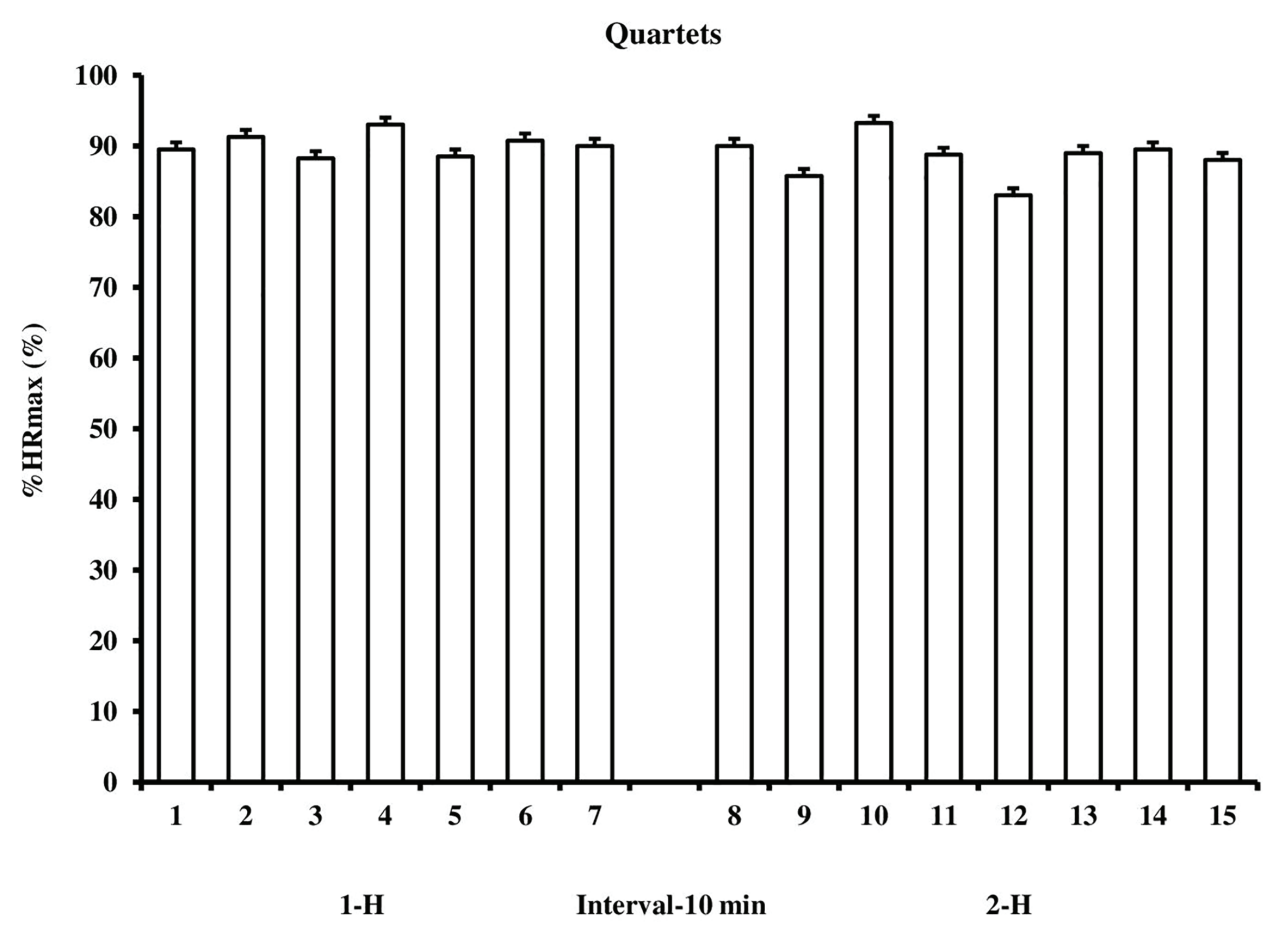
Percentage of maximum heart rate (%HR_max_), considering mean of quartets (four outfield players on the court) in first (1-H) and second (2-H) halves.

**Figure 2:**
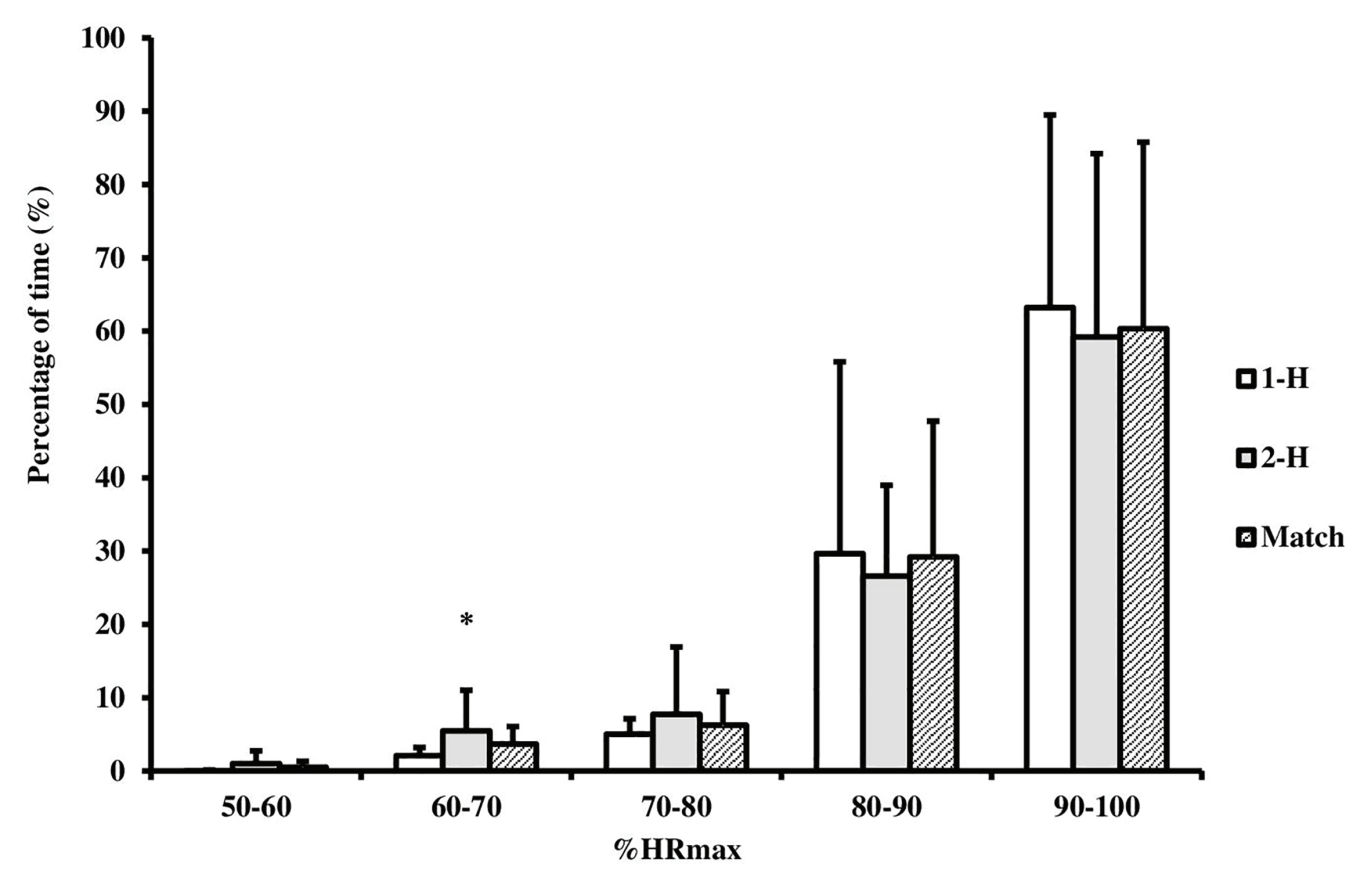
Percentage of time in five zones of %HR_max_. 1-H = first and 2-H = second halves, match = 1-H and 2-H. ^*^Significant difference between 1-H and 2-H (*p* = 0.02, *d* = 0.63, ES, moderate).

Players participated on average twice in each half, one player participated only once in each half, while in 1-H six players participated twice and in 2-H three players participated twice and three players were substituted three times. There were 12 substitutions in each half and the 24 substitutions originated 15 formations from four outfield players on the court (15 quartets, = 88.73 ± 2.49 %HR_max_), 7 quartets in 1-H (89.57 ± 1.62 %HR_max_, CI 95% = 88.07–91.07) and 8 in 2-H (88.00 ± 2.98 %HR_max,_ CI 95% = 85.51–90.49), no significant difference between 1-H and 2-H, *p* = 0.24, *d* = 0.66 (ES moderate). There was little variation in HR_mean_ among the four outfields players, quartets, CV% = 5 ± 4% in entire match, 4 ± 3% and 6 ± 4% in 1-H and 2-H, respectively, with only two quartets above 10% (11 and 12%) (Figure 1). Percentage time for which the players remained in each of the five %HR_max_ zone did not differ between 1-H and 2-H either, except 60–70 %HR_max_ in which players remained longer in 2-H (Figure 2): 50–60, *p* = 0.07, *r* = 0.49 (ES, moderate); 60–70, *p* = 0.02, *r* = 0.63 (ES, moderate); 70–80, *p* = 0.74, *r* = 0.09 (ES, trivial); 80–90, *p* = 0.74, *r* = 0.09 (ES, trivial) and 90–100% of HR_max_, *p* = 0.50, *r* = 0.18 (ES, small). Most of the match the players remained in the zone of higher intensity (> 90% HR_max_), 55.4 ± 26.3% in entire match, 57.2 ± 26.3% and 54.8 ± 26.3% in 1-H and 2-H, respectively.

In:Out_court_ ratio of the players in the entire match presented an average of 1:1.18 ± 1:0.51 min and there was no significant difference between 1-H and 2-H in In:Out_court_ (1:1.30 ± 1:0.48 vs. 1:1.05 ± 1:0.55 min, respectively), *p* = 0.64, *d* = 0.50 (ES, small). Total time, and of each player’s participation on the court also did not differ between 1-H and 2-H, although these parameters were lower in 1-H (Table 1).

## Discussion

The aimed of this study was to compare HR and [La^−^] responses between 1-H and 2-H, considering the time of permanency of the players on the court at each substitution, including the time of In:Out_court_ ratio and HR% from quartets outfield players on the court. Our results confirm our hypothesis that it is possible to maintain similar intensity of effort between the 1-H and 2-H of the match, since HR and [La^−^], both mean and maximum, did not differ between 1-H and 2-H (ES, trivial-small), nor did the time players remained in the five HR intensity zones, except to 60–70 %HR_max_ (ES, moderate).

In a simulated match of 4 × 10 min with 5 min of recovery, the HR_mean_ was not altered throughout the match (Castagna et al., 2009) and between the first and second half (Milioni et al., 2016). In contrast, Barbero-Alvarez et al. (2008) verified lower mean HR in the second half compared to the first half of official matches, 91.1 vs. 88.1% of HR_max_, respectively. However, players in that study covered the distance of 2,496 and 2,596 m, corresponding to 118 and 111 m·min^−1^ in the first and second half, respectively, approximately 44 min throughout the match, with increase of 12% in the time played in second half. Certainly, players covered a greater distance because they stayed more time on the court, but at lower speed. It is possible that excessive time on the court caused reduction of HR in the second half in that study of Barbero-Alvarez et al. (2008). In matches of the National Leagues of Brazil (Rodrigues et al., 2011) and Spain (Dogramaci et al., 2015), players remained 34 and 39 min on the court, respectively. In the present study, the players remained shorter time on the court (31 min) than players in the above mentioned studies (Barbero-Alvarez et al., 2008; Rodrigues et al., 2011; Dogramaci et al., 2015), and in the present study the players participated in average twice in each half, playing an average of 8 min each on the court. Thus, with a suitable time of 8 min in each participation and total time of 31 min in the match it was possible to maintain the intensity in the 2-H, whereas over 40 min on the court was not possible (Barbero-Alvarez et al., 2008). In addition, another interesting data analyzed in match was to the relationship between the time the players participated in the match and the time they were out of the match, resting (In:Out_court_ ratio). There was no difference in In:Out_court_ ratio between 1-H and 2-H. This is also another factor that may have contributed to the players maintaining similar effort intensity level between 2-H and 1-H. The literature has not addressed this In:Out_court_ ratio which needs to be further studied. In:Out_court_ ratio can be a parameter to be studied in future studies as it can have good applicability for coaches.

By stratifying HR in intensity zones it is possible to better analyze the effort intensity. Only in 60–70% of HR_max_ zone players spent more time during 2-H. On the other hand, time spent in three higher %HR_max_ zone (70–80, 80–90, and 90–100% of HR_max_) as well as in lower zone (50–60% of HR_max_) was similar between 1-H and 2-H. Time in lower intensity zones was not very significant for the match, since those two zones are the lowest intensity zones, corresponding to less than 5% of the total match time (see, Figure 2), which the players to use to recovery an actions less intensity. In contrast, Barbero-Alvarez et al. (2008) verified a reduction in players’ staying time above HR_max_ 85% in the second half of the match (86 to 79% of the time). As previously mentioned, the time a player remains on the court, certainly, can explain the difference between our results and the study by (Barbero-Alvarez et al., 2008).

In addition to the HR, blood samples were collected throughout the match for [La^−^] analysis at each player’s substitution. Although [La^−^] values were high, there was no reduction between 1-H and 2-H. In a simulated match (4 × 10 min/5 min pause), [La^−^] was not changed during the four periods of the match (Castagna et al., 2009), as well as in a friendly match (Arslanogu et al., 2014). On the other hand, [La^−^] responses were lower in 2-H in friendly matches of universities (Tessitore et al., 2008) and in amateur friendly match in 1-H and 2-H, respectively (Antunes Neto et al., 2007). However, the level of players (amateur) and the absence of substitutions during the match may explain reduction in [La^−^] in 2-H in these two studies. In others team sports, as basketball (Stojanovic et al., 2018) and soccer (Stolen et al., 2005) there is a reduction of [La^−^] between 1-H and 2-H, but with no evidence of [La^−^] reduction at the end of the handball match (Karcher and Buchheit, 2014).

Usually, blood collections in team sports are taken at the end of the first and second half of the match, or only at the end of the match. This can result in substantial loss of information (Stolen et al., 2005). We collected blood at each player’s substitution (average 8 min playing on the court), and despite the understanding of the limitation to study the response of [La^−^] in team sports, to our knowledge, this is the first study that has verified [La^−^] of all players’ participations in futsal match, which ensures a good representation of the [La^−^] response in futsal. The highest value of [La^−^]_mean_ (8.3 mmol·L^−1^) verify in a futsal match may be explained by high demand during the match (89% HR_max_ with CV% = 7%), which certainly hampered recovery between intense efforts. In addition, the higher level of the opponent may also have influenced the high demand of the evaluated team, as observed in soccer by Castellano et al. (2011), in relation to displacement in different intensity zones. The low intermittent aerobic fitness (YYIR-1 = 1,497 m) of the players is also a preponderant factor that can explain the high values of [La^−^], since the [La^−^] response is result of the production/removal ratio, which is influenced by aerobic fitness, among other factors (Stolen et al., 2005). However, despite the low aerobic fitness, playing with a higher quality opponent and the high [La^−^] in 1-H, the [La^−^] did not reduce in the 2-H.

To our understanding, the HR and [La^−^] values similar between the 1-H and 2-H can be explained through the temporal analysis of the players’ substitutions and the time on the court. Substitutions were made by the coach, without intervention from the researchers. There were 12 substitutions, players participated two times in each half, with no difference in time on the court between 1-H and 2-H as well as in the In:Out_court_ ratio, which certainly influenced the maintenance of the intensity in the 2-H of the match.

In soccer, the intensity of the match is 5–10% lower in the 2-H (Stolen et al., 2005; Dellal et al., 2012), certainly, due to the limited substitutions. Studies on substitutions in soccer have shown that the addition of players in 2-H attenuates the reduction of intensity (Mohr et al., 2003; Coelho et al., 2012; Bradley et al., 2014). In addition, Mohr et al. (2005) demonstrated that fatigue in soccer is temporary, considering intensity reduction after 5 min of intense activities. In futsal, with the possibility of unlimited substitutions, the drop in pace after a period of high intensity can be avoided by replacing one or more players. Reinforcing this idea, in studies on analysis of displacement during the match, de Oliveira Bueno et al. (2014) and Caetano et al. (2015) pointed that the decrease in physical performance or fatigue in futsal can be avoided by increasing the number of substitutions. Our results can also serve as a basis for other sports, such as basketball and handball, due to the similarity in unlimited substitutions, since in basketball HR and [La^−^] decay in the second half, and HR in the last quarter is lower than in the other quarters (Stojanovic et al., 2018), and in handball [La^−^] and HR responses tend to be similar and slightly lower, respectively (Karcher and Buchheit, 2014). Besides, contextual variables such as location at home or away, tactical system, level of physical fitness, quality of opponent, match status, losing, drawing or winning, among other factors can influence the results and need to be further studied in futsal, since in soccer, it has been shown that contextual variables can influence the performance of the players (Aquino et al., 2017, 2020).

Other interesting data of our study was the %HR_max_ analysis of the four outfield players who were on the court at each substitution and composed the quartets during the match. %HR_max_ from quartets did not differ between the 1-H and 2-H. CV% of HR among the four outfield players presented low variations throughout the match 1-H (4%) and 2-H (6%). Result of other studies also indicate that, independent of positions, there is certain uniformity in match demands, to data of repeated sprints (Caetano et al., 2015), muscle damage and inflammation (De Moura et al., 2013). To our knowledge, this is the first study that presents information from the quartet players in futsal, which makes it difficult to compare with the literature. Together, these results indicate that players need the similar level of physical fitness.

This study presents an innovative and useful approach considering the physiological demands related to time on the court, mainly in relation to the response of [La^−^], HR of the quartets, and also in the analysis of the intermittent activity of the players’ substitutions on the court, such as the In:Out_court_ ratio. However, study limitations should be considered, this is a case study, which analyzed only one match, as well as contextual variables were not considered in present study, and can influence the players’ performance. On the other hand, considering the high values of [La^−^], HR_mean_, % HR_max_ it is possible assume that players’ commitment was similar to that of an official match, since there was a high motivation of the players to confront a national league team with several players from the Brazilian national team, including the best futsal player in the world at that time.

## Conclusion

The study presents results not yet investigated in the literature, such as the temporal analysis of the substitutions and of [La^−^] each substitution of the players. Our results indicate that [La^−^]_mean_, HR_mean,_ time of stay in the three highest intensity zones of %HR_max_ (70–80, 80–90, and 90–100 %HR_max_) did not alter between 1-H and 2-H. HR_mean_ of the match was similar to previous studies and [La^−^]_mean_ was the highest observed in studies of futsal to the moment. %HR_max_ of outfield quartets presented low coefficient of variation throughout the match well as HR of each player. The temporal analysis of the substitutions collaborated for a better understanding of the changes in the physiological responses between 1-H and 2-H, as well as collecting blood samples at each substitution for [La^−^] analysis. Although this is a case study with only one match and this present limitations for generalizing the results, our results are a good guidance to coaches and for application in future studies. Considering that the players did not present a decrease in the HR and [La^−^] responses in 2-H and analyzing the match’ temporal variables, we suggest that coaches balance the time on the court and the In:Out_court_ ratio of players in both halves of the match, with less participation in the first half, an average of 2–3 substitutions for each outfield player in each half of the match, duration of ~8 min for each players’ participation, and that the total playing time on the court is not much higher than 30 min.

## Data Availability Statement

The raw data supporting the conclusions of this article will be made available by the authors, without undue reservation, requesting the corresponding author.

## Ethics Statement

The studies involving human participants were reviewed and approved by Ethics Committee of the School of Science of the São Paulo State University. The patients/participants provided their written informed consent to participate in this study.

## Author Contributions

The study was designed by JD-S in collaboration with MP and PS who oversaw all procedures and the research phase. All other authors also participated in the discussion and preparation of the drawing. HS, RB, RS, FM, LV, and JD-S performed the data collection, and the data were tabulated and analyzed by OS, MP, DC, and LV. After that, the data were discussed by all the authors together who also collaborated with bibliographic suggestions. After the final editing by JD-S, the text was reviewed by all authors and the suggestions sent, and incorporated into the text for the final version. The final version was sent to everyone for approval before submission to the Journal.

### Conflict of Interest

The authors declare that the research was conducted in the absence of any commercial or financial relationships that could be construed as a potential conflict of interest.
